# PAG-dependent defensive strategy selection: From stress-induced rigidity to adaptive flexibility

**DOI:** 10.1016/j.isci.2026.115927

**Published:** 2026-04-29

**Authors:** Lianli Qiu, Yueqin Liu, Rongfeng Qi, Zhiqiang Zhang, Xiaoqing Cheng, Jianrui Li, Jing Pan, Wei Wu, Longjiang Zhang, Guangming Lu

**Affiliations:** 1 Department of Radiology, Jinling Clinical Medical College, Nanjing Medical University, Nanjing, Jiangsu 210002, China; 2 Department of Radiology, Jinling Hospital, Medical School, Nanjing University, Nanjing, Jiangsu 210002, China; 3 Department of Radiology, Nanjing Drum Tower Hospital, Affiliated Hospital of Medical School, Nanjing University, Nanjing, Jiangsu 210002, China

**Keywords:** defensive behavior flexibility, tone intensity, periaqueductal gray, chemogenetics, single prolonged stress

## Abstract

Adaptive survival requires flexibly choosing passive or active defenses as threat intensity varies. Many studies have described the execution of defensive behaviors, but the flexibility of mice to choose defensive strategies is poorly understood. Here, we demonstrate that auditory cues of different intensities (60 dB and 80 dB) elicit passive (freezing) and active (escape) defensive behaviors in mice, mimicking the flexibility to choose appropriate defensive behaviors when faced with varying degrees of threat. Single prolonged stress (SPS) mice exhibited dysregulated defensive behavior selection, showing a maladaptive tendency to freeze when facing imminent threats. Chemogenetic inhibition of the dorsal periaqueductal gray (dPAG) and ventrolateral periaqueductal gray (vlPAG) coordinates selective shifts in defensive behavior. These results imply that interactions between the dPAG and vlPAG may support flexible defensive strategy selection and influence behavioral output, highlighting PAG circuitry as a candidate target for improving adaptive responses in stress-related disorders.

## Highlights

• Auditory cue intensity biases selection of active and passive defensive responses
• SPS biases active behavior toward passive responding under high threat
• PAG subregions regulate flexible defensive strategy selection

## Graphical abstract

**Figure:**
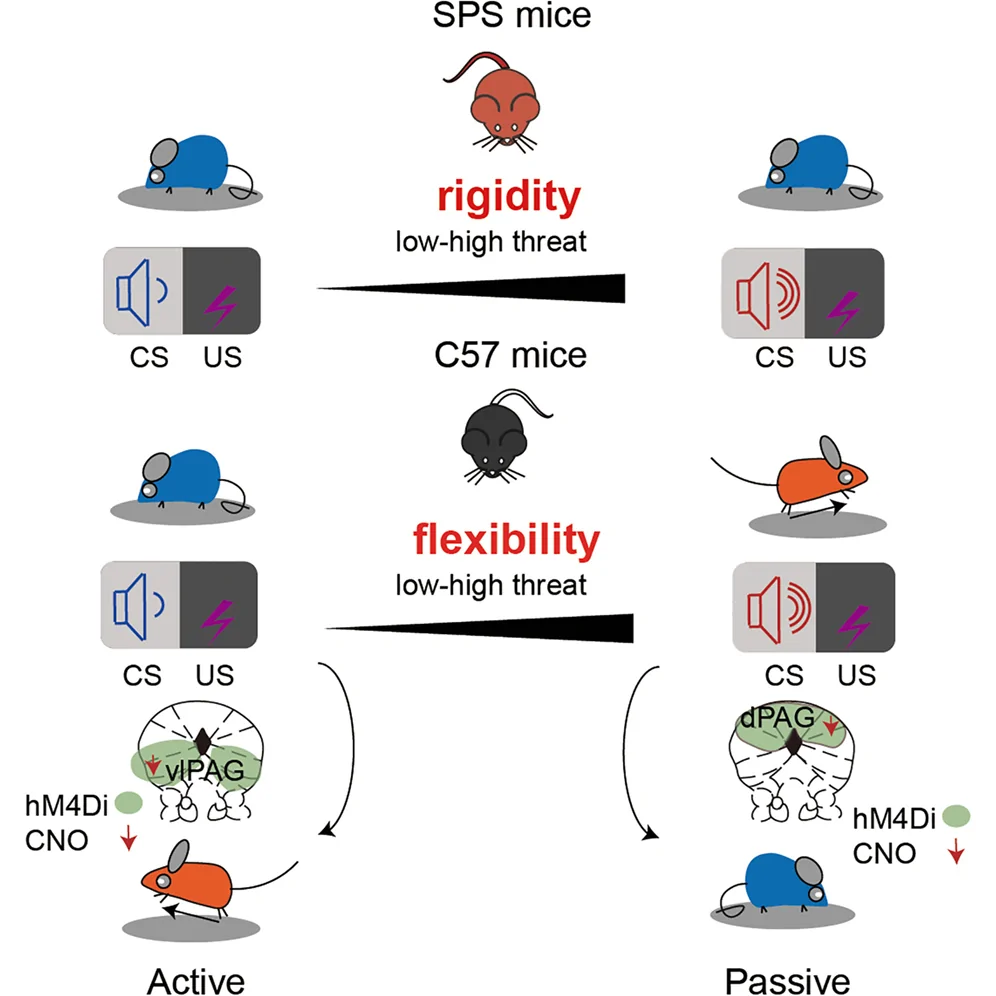

## Teaser

neuroscience; behavioral neuroscience; systems neuroscience

## Introduction

Survival in a dynamic and potentially threatening environment requires animals to quickly select appropriate defensive behavior.^1^ In rodents, defensive behaviors are broadly categorized into active strategies (e.g., flight or escape) and passive strategies (e.g., freezing or immobility), and animals could switch their distinctive responses depending on the perceived level of threat, the context of exposure, and previous experience with stimuli.^1^ In naturalistic settings, the concept of threat imminence typically refers to physical proximity or approach dynamics of a danger source (e.g., predator distance), which can strongly bias defensive action selection. The looming test, which presents an expanding disk to mimic an approaching predator, is commonly used to study innate defensive responses under naturalistic threat imminence.^2^^,^^3^ In contrast, threat is learned and associated with specific cues in conditioned fear paradigms. Variations in the cue are expected to alter sensory salience and may modulate the perceived threat level associated with the cue, rather than directly manipulating or measuring naturalistic imminence. Auditory tones are one of the most commonly used cues in conditioned fear learning paradigms. In natural settings, auditory-related cues also serve as crucial indicators of potential threats, guiding defensive behavior selection, particularly during combat scenarios or natural disasters. Previous studies have demonstrated that a serial compound stimulus can elicit distinct defensive behaviors in mice, such as freezing or escape responses.^4^ These findings suggest that auditory tone-based conditioned fear learning provides a reliable and flexible experimental framework, and the flexible neural mechanisms underlying auditory-induced learned defensive behaviors warrant further investigation.

To cope with varying threat levels, rodents must adopt rapid and flexible response selection, which is regulated by a distributed neural circuit across the prefrontal cortex,^5^ the central amygdala,^6^ hypothalamus,^7^ and midbrain.^8^ Within the midbrain, the periaqueductal gray (PAG) could integrate multiple inhibitory and excitatory inputs from upstream brain areas to mediate context-specific escape or freezing.^8^^,^^9^^,^^10^^,^^11^ Importantly, these computations are thought to be shaped by intrinsic GABAergic microcircuits within and across PAG columns, which gate the recruitment of downstream output ensembles and facilitate switching between active and passive defensive models.^8^^,^^12^^,^^13^ Functionally, the PAG exhibits a dorsoventral organization,^14^ in which the ventrolateral PAG (vlPAG) glutamatergic output neurons are required for normal freezing expression and can be sufficient to elicit freezing, whereas excitatory neurons in the dorsal PAG (dPAG) govern the initiation of escape responses via upstream inputs from the medial superior colliculus.^9^ Maladaptive outcomes arising from this flexible selection process are a hallmark of anxiety-related psychiatric disorders.^15^^,^^16^^,^^17^^,^^18^^,^^19^ Notably, repetitive stress can modulate sensory processing and the perception of neutral sounds in coordinating individuals’ psychological resilience in the face of stress.^20^^,^^21^ In this context, the PAG serves both as a key downstream target of auditory cortical projections that can elicit defensive responses, and as a locus of dysregulation implicated in stress-related psychiatric disorders.^11^^,^^22^^,^^23^^,^^24^ Therefore, further evidence is required to clarify the mechanisms by which interactions among PAG subregions regulate the flexible switch between passive and active defensive strategies in response to threats of varying intensities and determine how these subregions contribute to the dysregulation of defensive decision-making in stress-exposed mice.

Here, we utilized auditory stimuli of different intensities to simulate threat proximity, inducing distinct defensive behaviors in mice. The auditory stimuli of different intensities not only enable active-passive behavioral switching under controlled threat conditions but also reveal their alterations in stress-induced models such as SPS. The correlation between PAG subregions and defensive behavior switching may explain the flexible selection of active and passive defensive behaviors in mice. Moreover, the behavioral shifts in SPS mice also provide a valuable approach for probing the neural mechanisms underlying maladaptive defensive responses in psychiatric disorders.

## Results

### Active and passive conditioned defensive responses were evoked by auditory stimuli of different intensities

To investigate whether stimuli of low- and high-intensity can induce different defensive responses in mice, we used the classical Pavlovian fear conditioning experimental paradigm with stimuli of different intensity (Figure 1A). After habituating to the conditioning chamber on Day0, mice were exposed to three CS (30 s CS, CS: conditioned stimulus, 80 dB or 60 dB tone) to allow habituation to the auditory stimulus and five trials (29 s CS + 1 s US, US: unconditioned stimulus, 0.6 mA, shock) to induce tone-cued conditioned fear on day 1. During the tone habituation period, low-intensity tone (60 dB) mildly increased freezing levels in mice without altering their movement speed (Figures 1C–1E, BL). In contrast, mice exposed to high-intensity tone (80 dB) exhibited a sharp increase in speed at the onset of the stimulus, while their average movement speed during the tone presentation decreased, accompanied by elevated freezing levels (Figures 1C–1E, BL). This pattern indicates that high-intensity tones evoked a heightened state of alertness. The baseline responses to different intensities of tones reflect that high-intensity tones could represent a perceived higher level associated with the cue.

**Figure 1:**
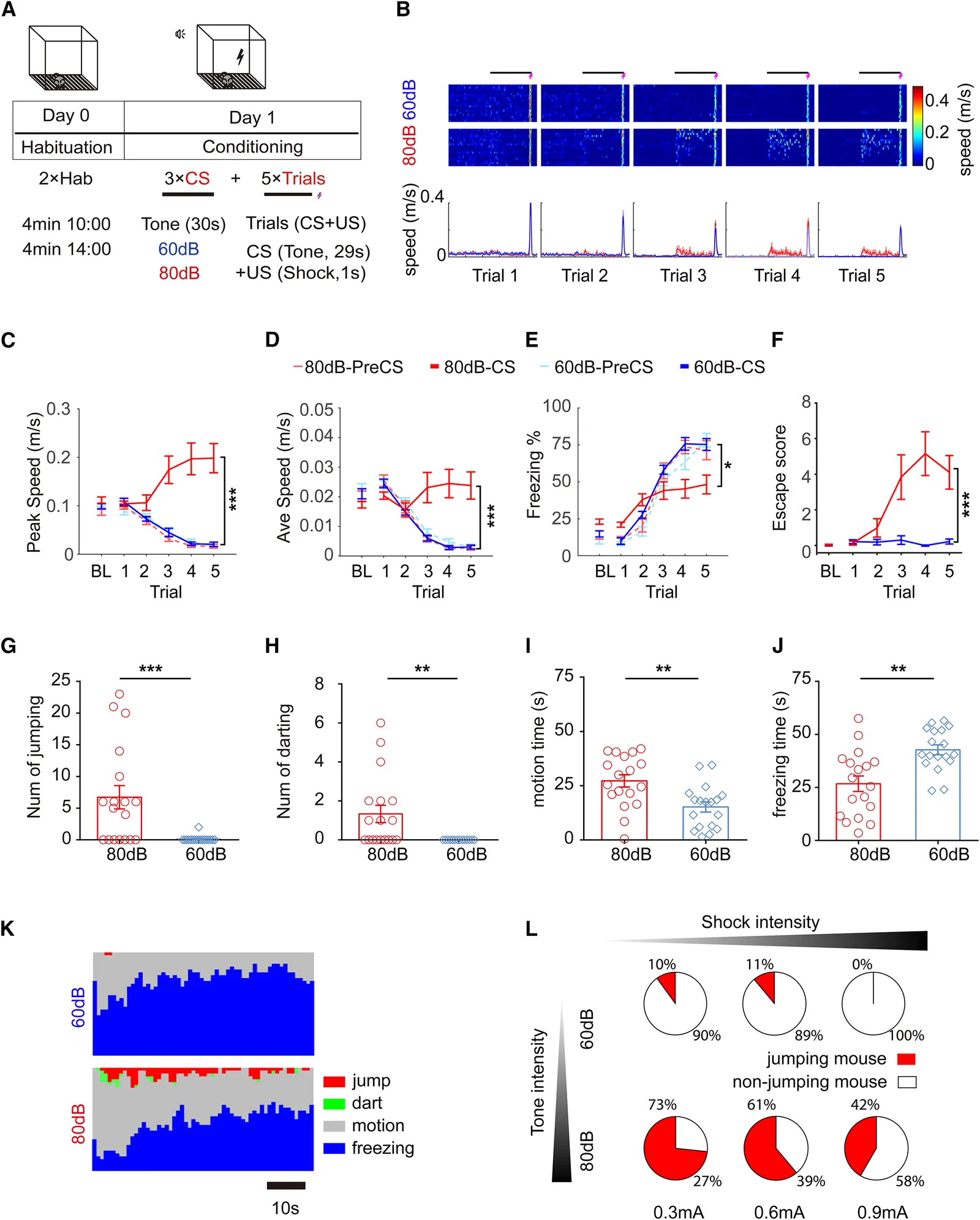
Active and passive conditioned defensive responses were evoked by auditory stimuli of different intensities (A) Schematic of the experimental paradigm (CS, conditioned stimuli, 60 dB and 80 dB; US, unconditioned stimuli, 0.6 mA). Two independent groups of mice were conditioned with different CS intensities (*n* = 18 mice per group). (B) Heatmap (top) and speed traces (bottom) from all mice showing movement speed during trial 1–5 in the 60 dB group (*n* = 18 mice) and the 80 dB group (*n* = 18 mice). (C–F) Quantification of peak speed, average speed, freezing percentage and escape score during baseline (BL: mean across the three tone habituation period on Day1) and five consecutive trials (trial 1–5, day 1) in the 60 dB and 80 dB groups. Normality was assessed using Shapiro-Wilk tests (Table S1). Two-way repeated-measures ANOVA (Trial × dB) revealed significant Trial × dB interactions for peak speed (F[4, 136] = 17.20), average speed (F[4, 136] = 13.76), freezing percentage (F[4, 136] = 16.89), and escape score (F[4, 136] = 7.885) (all *p* < 0.0001), with significant main effects of dB (peak speed, F[1, 34] = 32.12, *p* < 0.0001, average speed, F[1, 34] = 15.78, *p* = 0.0004, freezing percentage, F[1, 34] = 5.302, *p* = 0.0275, escape score, F[1, 34] = 18.75, *p* = 0.0001). Bonferroni-corrected simple-effects comparisons are provided in Table S2. (G–J) Behavioral metrics of jumping, darting, motion, and freezing times were manually quantified during the last two CS presentations at two distinct tone intensities (60 dB vs. 80 dB; *n* = 18 mice per group, same cohorts as in B–F). Normality was assessed using Shapiro-Wilk tests (Table S3). Jumping and darting were compared using Mann-Whitney U tests (jump, *p* < 0.001, dart, *p* = 0.001), and motion and freezing times were compared using two-tailed unpaired *t* tests (motion time, *p* = 0.002, freezing time, *p* = 0.001, Welch’s correction was applied for freezing due to unequal variances). (K) The time course of conditioned behavioral metrics at two distinctive tone intensities. (L) Proportion of jumping and non-jumping mice in response to different tone intensities (60 dB and 80 dB) and footshock intensities (0.3 mA, 0.6 mA, and 0.9 mA). In addition to the 0.6 mA cohorts shown in (A–K), additional cohorts were tested at 0.3 mA and 0.9 mA. Pie charts indicate the percentage of mice exhibiting jumping behavior under each condition. (0.3 mA, 60 dB, *n* = 10 mice, 80 dB, *n* = 15 mice, 0.9 mA, 60 dB, *n* = 10 mice, 80 dB, *n* = 12 mice). Data are presented as mean ± SEM, ∗*p* < 0.05, ∗∗*p* < 0.01, ∗∗∗*p* < 0.001.

Within the following fear conditioning training, mice both exhibited progressively increased levels of freezing in different intensity tone exposures. Consistent with the previous study,^25^^,^^26^ mice trained with low-intensity CS developed a passive defensive behavior, demonstrated by gradually decreased speed and increased freezing levels (Figures 1B–1E). In contrast, the mice trained with high-intensity CS exhibited active defensive behavior, with gradually increased movement speed and a freezing level that steadily increased at a slower rate compared to the low-intensity CS presentation (Figures 1B–1E). However, in the session that received only CS presentations, the 80 dB stimulus produced markedly more freezing than the 60 dB stimulus, yet overall freezing remained at baseline levels comparable to the habituation period (Figure S1). Furthermore, a passive defensive response was evoked by distinctive tone intensities during fear memory retrieval (Figure S2). These results indicated that mice learned to adopt distinctive defensive behavior in response to different tone intensities that likely modulate the perceived threat level or salience and thus approximate different threat states.

Furthermore, we specified the behavioral metrics to evaluate the defensive response of mice, including jumping, darting, motion, and freezing in the last two CS presentations. Compared to mice exposed to low-intensity CS, those with high CS exhibited more jumping and darting, increased movement time, and reduced freezing time (Figures 1G–1J). Collectively, mice with high-intensity CS show a higher escape score than the low-intensity CS during the conditioning (Figure 1F). Though there is a sudden increase in motion at the beginning of the high-intensity tone presentation, the jump and dart were uniformly distributed during the high-intensity tone presentation (Figure 1K). These suggest that active behavior is not a panic response to the tone, but a developing defensive strategy to imminent danger. To test the effect of shock intensity on the defensive responses, we added two additional groups of shock intensities: 0.3 mA and 0.9 mA. Mice that exhibited jumping behavior during the conditioned tone presentations were defined as jumping mice. Only a small portion of mice exhibited active defensive behavior in response to low CS presentation, whereas high-intensity tone induced a higher proportion of mice displaying jumping behavior (Figure 1L). Although this relationship was influenced by shock intensity and was not strictly linear, these results still indicate an overall pattern of defensive behavior selection in response to different perceived threat levels associated with the cue. Based on the non-linear response pattern observed at higher shock intensities, 0.6 mA was selected for the subsequent stress-exposure experiments because it reliably elicited jumping while avoiding the more complex behavioral effects observed at 0.9 mA.

### SPS mice exhibited stress-induced rigidity in defensive behavior when faced with a high-intensity threat

To examine whether traumatic stress impairs the adaptive selection of defensive behaviors under different perceived threat levels, we employed the single prolonged stress (SPS) paradigm to model post-traumatic stress disorder (PTSD)-like symptoms in mice.^27^^,^^28^ Fifteen days after stress exposure, animals underwent the basic analysis of the open field test (OFT) and elevated plus maze (EPM). We found a decrease in total distance and mean speed in the SPS mice (Figure S3B). The OFT results for SPS mice also exhibited decreased measures of center entry, center time, and center distance compared with the control group (Figure S3C). Relative to the control mice, the SPS mice also showed a reduction in open-arm entries and open-arm time (Figure S3D) for the EPM, which reflects increased anxiety of SPS mice. These results demonstrate that this model can lead to symptoms of anxiety and decreased movement in mice.

To demonstrate how stress influences the defensive strategy selection, animals underwent auditory threat conditioning with tones of either low (60 dB) or high (80 dB) intensity 15 days after stress exposure (Figure 2A). During the habituation period, SPS mice exhibited comparable movement speed and freezing levels in response to both low- and high-intensity tones (Figures 2C–2E and S4B–S4E, BL). Under low-tone intensity conditions, SPS mice exhibited similar freezing levels and locomotor speed compared to the control mice (Figures 2B and S4), suggesting that the traumatic stress could not impact the passive defensive responses when faced with low-tone intensity threat. However, when exposed to high-tone intensity (80 dB), locomotor speed alterations emerged (Figure 2B). During the conditioning trials, SPS mice displayed significantly higher freezing levels and reduced average and peak locomotor speed compared with controls (Figures 2C–2E). To further assess active versus passive behavioral patterns, we quantified specific defense responses during the last two CS presentations. SPS mice showed significantly fewer jumping and darting (Figures 2G and 2H), along with shorter movement time and prolonged freezing duration (Figures 2I and 2J) compared with controls. Moreover, SPS mice exhibited significantly lower escape scores than the control mice, indicating a shift from active to passive defensive strategies in response to the higher-perceived threat (Figure 2F). These results demonstrate that prior stress exposure biases mice toward passive defensive responses when confronted with high-intensity threats, reflecting impaired behavioral flexibility and potentially reduced adaptive value in survival contexts.

**Figure 2:**
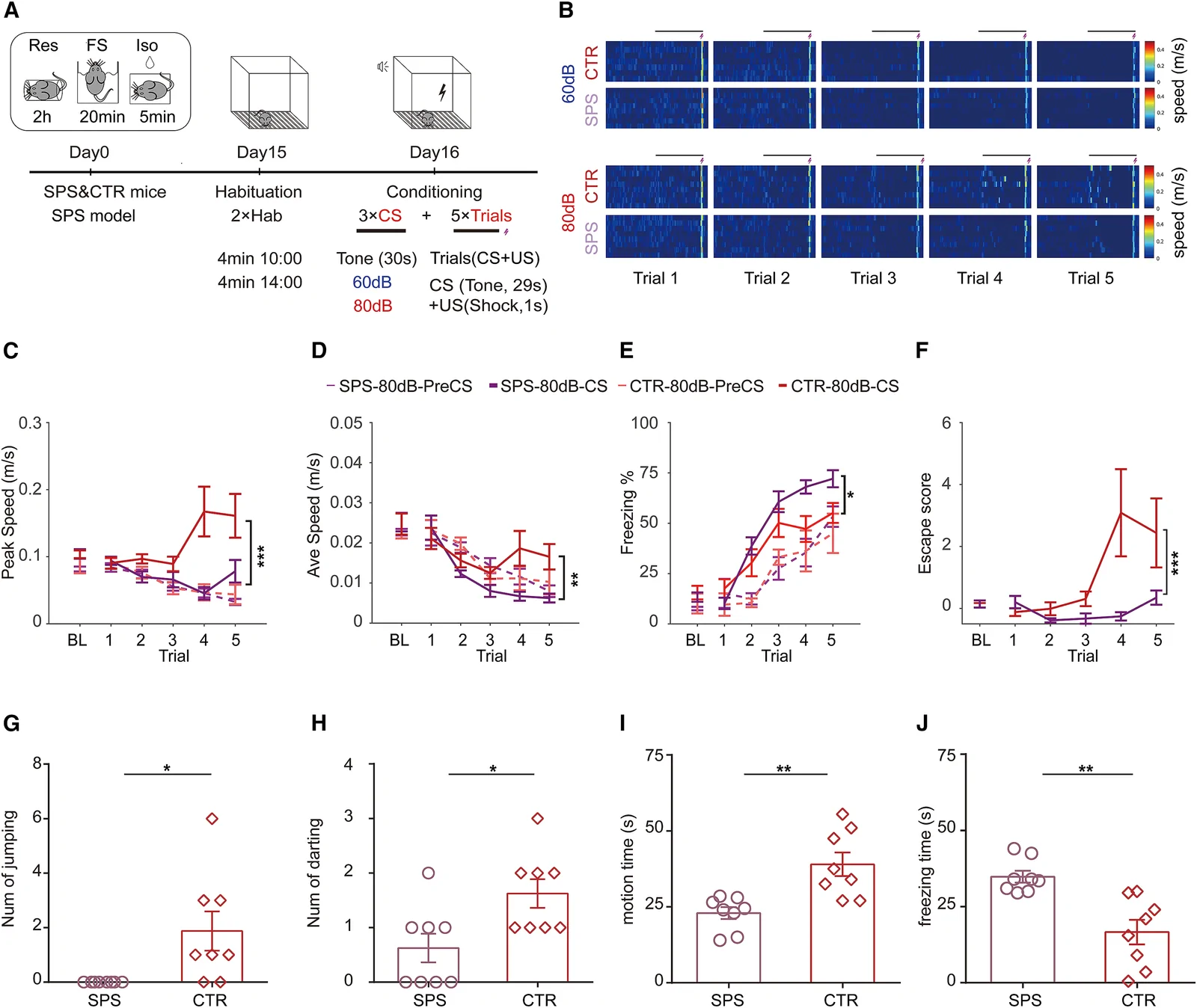
SPS mice exhibited stress-induced rigidity in defensive behavior when faced with a high-intensity threat (A) Experimental design for the SPS and control mice exposed to the different tone intensity fear conditioning (CS, 60 dB and 80 dB, 29 s; US, 0.6 mA, 1 s). (B) Heatmaps of SPS and control mice showing movement speed during trial 1–5 in the 60 dB group (SPS, *n* = 8 mice, CTR, *n* = 7 mice) and the 80 dB group (SPS, *n* = 8 mice, CTR, *n* = 8 mice). (C–F) Quantification of peak speed, average speed, freezing percentage, and escape score during baseline (BL: mean across the three tone habituation period on day 16) and five consecutive trials (trial 1–5) exposed to 80 dB in SPS and control mice (*n* = 8 mice per group). Normality was assessed using Shapiro-Wilk tests (Table S1). Two-way repeated-measures ANOVA (Trial × SPS) revealed significant Trial × SPS interactions for peak speed (F[4, 56] = 4.374, *p* = 0.0038), average speed (F[4, 56] = 3.507, *p* = 0.0126), freezing percentage (F[4, 56] = 2.608, *p* = 0.0452), and escape score (F[4, 56] = 2.795, *p* = 0.0346), with significant main effects of SPS (peak speed, F[1, 14] = 24.92, *p* = 0.0002, average speed, F[1, 14] = 16.06, *p* = 0.0013, freezing percentage, F[1, 14] = 5.985, *p* = 0.0282, escape score, F[1, 14] = 0.52, *p* = 0.0005). Bonferroni-corrected simple-effects comparisons are provided in Table S2. (G–J) Behavioral metrics of jumping, darting, motion, and freezing times were manually quantified during the last two CS presentations of SPS and CTR mice (*n* = 8 mice per group). Normality was assessed using Shapiro-Wilk tests (Table S3). Jumping and darting were compared using Mann-Whitney U tests (jump, *p* = 0.035, dart, *p* = 0.022), and motion and freezing times were compared using two-tailed Welch’s *t* tests (motion time, *p* = 0.004, freezing time, *p* = 0.002). Data are presented as mean ± SEM, ∗*p* < 0.05, ∗∗*p* < 0.01, ∗∗∗*p* < 0.001.

### Chemogenetic inhibition of dPAG shifts active to passive defensive responses during high-intensity fear conditioning

The PAG exhibits an important role in defensive responses. Activation of vlPAG neurons using optogenetic techniques triggers robust freezing aimed at avoiding predators, and neurons in the dPAG encode escape behavior of innate defensive responses.^8^^,^^9^ To investigate whether these two subregions are involved in distinct defensive responses, we compared the expression of c-Fos protein in C57 and SPS mice after conditioning to respective tones (Figure 3A). Compared with C57 mice exposed to a low-intensity tone, those exposed to a high-intensity tone exhibited a greater number of c-Fos-positive neurons in the dPAG, whereas the number of vlPAG neurons was similar in both groups (Figure 3B; Table S3). SPS mice showed significantly fewer c-Fos-positive neurons in the dPAG than C57 mice when exposed to high-intensity condition, and vlPAG c-Fos labeling did not differ appreciably between SPS and C57 mice (Figures 3A and 3B). High-tone exposure was associated with increased dPAG c-Fos-positive neurons, which coincided with a shift toward more active defensive behaviors, consistent with greater recruitment of dPAG neurons under higher perceived threat. Furthermore, stress exposure blunted dPAG responses to the high-intensity condition, consistent with reduced recruitment of dPAG neurons under high-intensity conditions.

**Figure 3:**
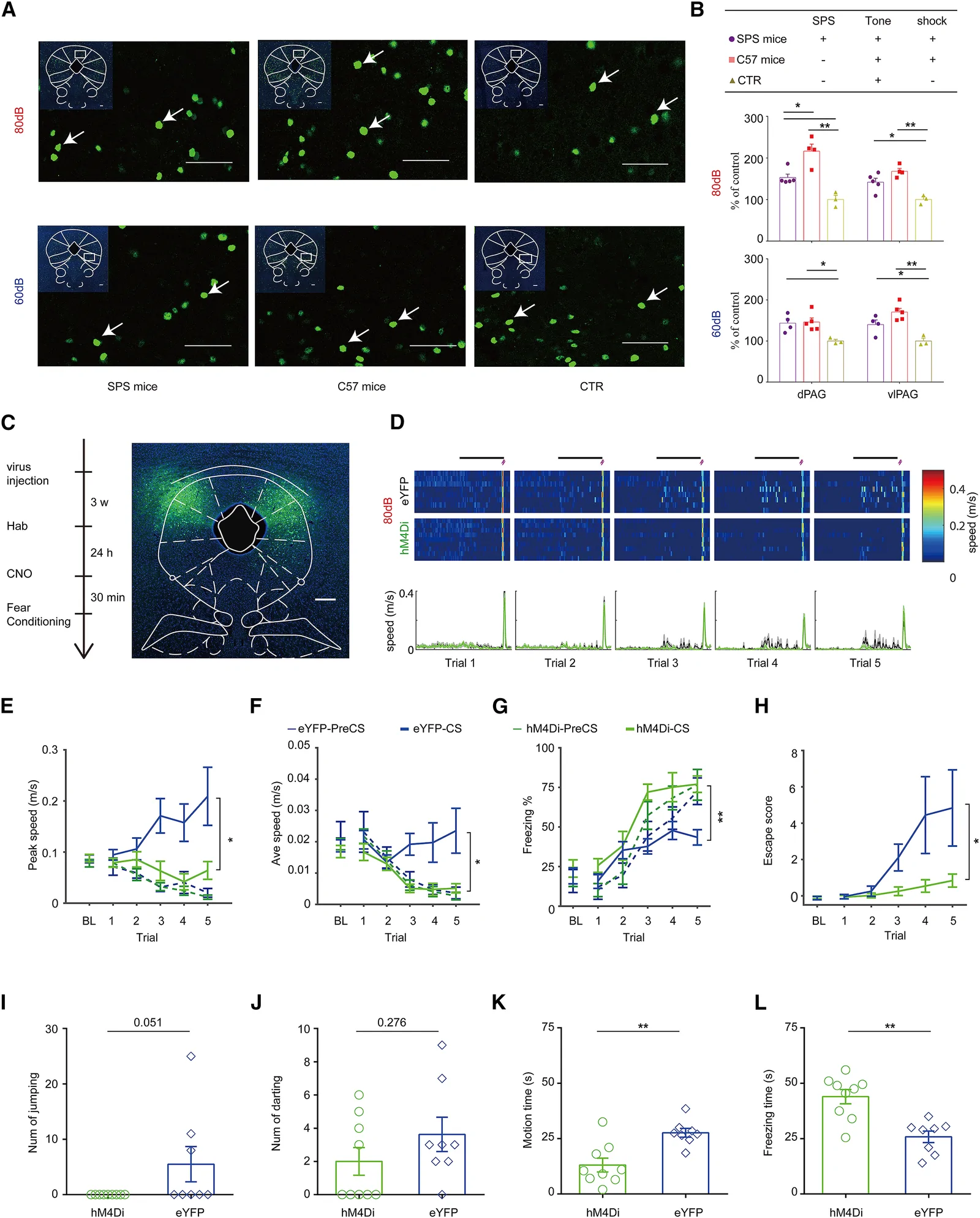
Chemogenetic inhibition of dPAG shifts active to passive defensive responses during high-intensity fear conditioning (A) Representative c-Fos staining in different groups (Arrows indicate Fos-positive nuclei.). Scale bars, 100 μm. (B) Quantification of c-Fos positive cells in the dPAG and vlPAG under the low and high intensity conditions across SPS, C57, and control mice (80 dB, SPS mice, *n* = 5 mice, C57 mice, *n* = 4 mice, high-tone CTR, *n* = 3 mice, 60 dB, SPS mice, *n* = 4 mice, C57 mice, *n* = 5 mice, low-tone CTR, *n* = 3 mice). Normality was assessed using Shapiro-Wilk tests. For pairwise group comparisons, two-tailed unpaired *t* tests were used when normality assumptions were met, otherwise, two-tailed Mann-Whitney *U* tests were applied. The normality and pairwise group comparison of different groups was seen in Table S3. (C) Experimental design for the high tone intensity fear conditioning. Left: schematic of experimental paradigm (CS, 80 dB, US, 0.6 mA). Right: representative expression of hM4Di virus in the dPAG. Scale bars, 200 μm. (D) Heatmaps (top) and speed traces (bottom) from all mice showing movement speed during trial 1–5 for eYFP group (*n* = 8 mice) and hM4Di group (*n* = 9 mice). (E–H) Quantification of peak speed, average speed, freezing percentage, and escape score during baseline and five consecutive trials in eYFP group (*n* = 8 mice) and hM4Di groups (*n* = 9 mice). Normality was assessed using Shapiro-Wilk tests (Table S1). Two-way repeated-measures ANOVA (Trial × hM4Di) revealed significant Trial × hM4Di interactions for peak speed (F[4, 60] = 5.018, *p* = 0.0015), average speed (F[4, 60] = 4.079, *p* = 0.0054), freezing percentage(F[4, 60] = 4.367, *p* = 0.0036), and escape score (F[4, 60] = 2.876, *p* = 0.0302), with significant main effects of hM4Di (peak speed, F[1, 15] = 7.962, *p* = 0.0129, average speed, F[1, 15] = 8.063, *p* = 0.0124, freezing percentage, F[1, 15] = 12.18, *p* = 0.0033, escape score, F[1, 15] = 5.489, *p* = 0.0333). Bonferroni-corrected simple-effects comparisons are provided in Table S2. (I–L) Behavioral metrics of jumping, darting, motion, and freezing times were quantified during the last two CS presentations of the eYFP and hM4Di groups. Normality was assessed using Shapiro-Wilk tests (Table S3). Jumping and darting were compared using Mann-Whitney U tests (jump, *p* = 0.051, dart, *p* = 0.276), and motion and freezing times were compared using two-tailed unpaired *t* tests (motion time, *p* = 0.002, freezing time, *p* = 0.001). Data are presented as mean ± SEM, ∗*p* < 0.05, ∗∗*p* < 0.01.

We have found that the mice would adopt active defensive responses in the high tone intensity, and then we applied the chemogenetics to examine whether dPAG neurons’ activity could transform the choice of active defensive responses. We targeted dPAG neurons by local injection of rAAV-hSyn-hM4Di-eYFP, and the control mice were injected with AAVs only containing eYFP fluorescent protein tags (Figure 3C). The extent of viral infection was outlined in the subregion of dPAG matched to the brain atlas (Figure S5A). Mice were administered CNO 30 min before fear conditioning (Figure 3C). During the tone habituation period, chemogenetic inhibition of the dPAG brain region does not alter the baseline activity of hM4Di mice in response to high-intensity tone compared to the eYFP group (Figures 3E–3G, BL). As shown in the heatmaps and speed traces, the eYFP group exhibited robust speed increases and escape-like behavior in response to the high-tone intensity fear conditioning (Figure 3D). In contrast, hM4Di-expressing mice showed diminished active defensive responses. The hM4Di-expressing mice converted a gradually increased speed and showed a higher rate of increased freezing levels compared to the eYFP group (Figures 3E–3G). The behavioral metrics of defensive responses revealed that the hM4Di group exhibited less jumping and darting, movement time, and more freezing time (Figures 3I–3L). Collectively, the hM4Di mice showed a lower escape score than the eYFP groups during the high-tone intensity fear conditioning (Figure 3H). Together, these results demonstrated the critical role of the dPAG in orchestrating threat-appropriate behavioral output in the face of a higher perceived threat level.

### Chemogenetic inhibition of vlPAG shifts passive to active defensive responses during low-intensity fear conditioning

We have demonstrated that inhibiting dPAG neurons can shift active to passive defensive behaviors. The vlPAG is a brain region associated with freezing behavior. Furthermore, we explored the changes in freezing behavior by inhibiting the activity of vlPAG neurons (Figure 4A). The extent of viral infection was outlined in the vlPAG matched to the brain atlas (Figure S5B). And the CNO was also intraperitoneally injected to inactivate the vlPAG neurons (Figure 4A). Chemogenetic inhibition of the vlPAG brain region increased movement speed and reduced freezing levels throughout the tone habituation period, suggesting that inhibition of the vlPAG region mildly elevated baseline activity levels (Figures 4C–4E, BL). Within the following low-intensity tone paired with shock, the hM4Di-expressing mice also showed higher movement speed compared to the eYFP group (Figures 4C and 4D). While the hM4Di group did not exhibit the increased speed like the high-intensity tone group (Figures 1C and 1D, 80 dB group), their movement speed and escape score were still higher than those of the eYFP group (Figures 4C, 4D, and 4F). And the hM4Di-expressing mice exhibited a steady increase in freezing behavior, though at a slower rate compared to the eYFP group (Figure 4E). Although chemogenetic inhibition of the vlPAG did not induce any jumping behavior in response to the low-intensity tone, behavioral metrics of the hM4Di group showed more darting and motion time compared to the eYFP group, resulting in less freezing time (Figures 4G–4J). Taken together, the more active defensive behaviors in the vlPAG hM4Di group illustrated that chemogenetic inhibition of vlPAG converts passive defensive responses to active responses to low-intensity tone. Chemogenetic manipulations of distinct PAG subregions reveal a dynamic interplay between active and passive defensive responses, indicating that under conditions of relatively perceived mild threat, the vlPAG population predominates, whereas with perceived higher threat level, dominance shifts to dPAG populations.

**Figure 4:**
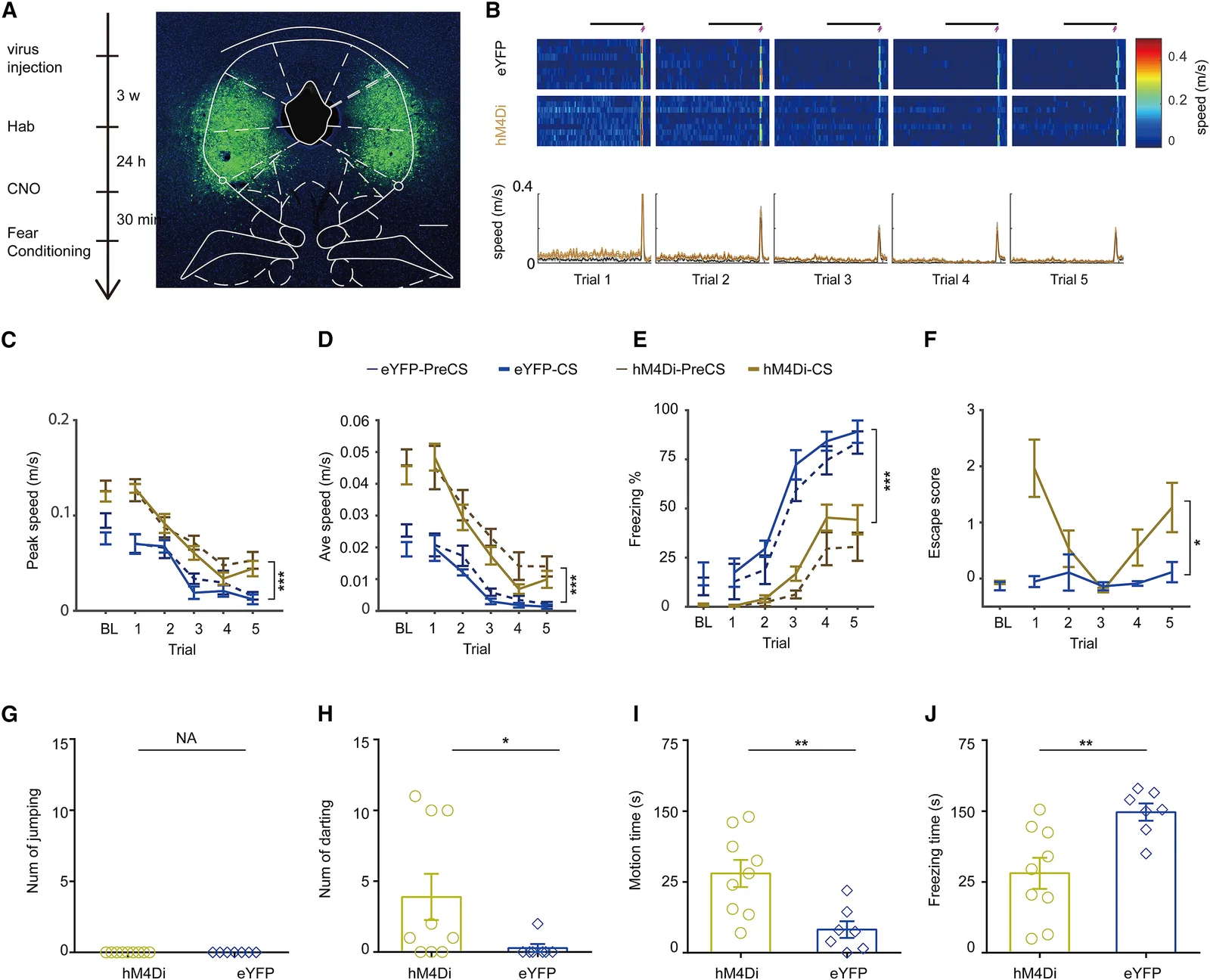
Chemogenetic inhibition of vlPAG shifts passive to active defensive responses during low-intensity fear conditioning (A) Experimental design for the low tone intensity fear conditioning. Left: schematic of experimental paradigm (CS, 60 dB, US, 0.6 mA). Right: representative expression of hM4Di virus in the vlPAG. Scale bars, 200 μm. (B) Heatmaps (top) and speed traces (bottom) from all mice showing movement speed during trial 1–5 for eYFP (*n* = 7 mice) and hM4Di (*n* = 9 mice) groups. (C–E) Quantification of peak speed, average speed, freezing percentage, and escape score during baseline and five consecutive trials in the eYFP group (*n* = 7 mice) and hM4Di group (*n* = 9 mice). Normality was assessed using Shapiro-Wilk tests (Table S1). Two-way repeated-measures ANOVA (Trial × hM4Di) revealed significant Trial × hM4Di interactions for peak speed (F[4, 56] = 3.124, *p* = 0.0217), average speed (F[4, 56] = 6.777, *p* < 0.0001), freezing percentage (F[4, 56] = 7.570, *p* < 0.0001), and escape score (F[4, 56] = 2.883, *p* = 0.0306), with significant main effects of hM4Di (peak speed, F[1, 14] = 26.72, *p* = 0.0001, average speed, F[1, 14] = 27.72, *p* = 0.0001, freezing percentage, F[1, 14] = 81.88, *p* < 0.0001, escape score, F[1, 14] = 7.755, *p* = 0.0146). Bonferroni-corrected simple-effects comparisons are provided in Table S2. (G–J) Behavioral metrics of darting, motion, and freezing times were manually quantified during the last two CS presentations of the eYFP and hM4Di groups. Normality was assessed using Shapiro-Wilk tests (Table S3). Darting was compared using Mann-Whitney U tests (jump, all values = 0, dart, *p* = 0.047), and motion and freezing times were compared using two-tailed unpaired *t* tests (motion time, *p* = 0.006, freezing time, *p* = 0.005, Welch’s correction was applied for freezing due to unequal variances). Data are presented as mean ± SEM, ∗*p* < 0.05, ∗∗*p* < 0.01, ∗∗∗*p* < 0.001.

## Discussion

Our study reveals that mice are capable of behavioral flexibility in defensive responses based on two different tone intensities during fear conditioning, despite exhibiting predominantly passive responses during conditioned stimulus retrieval. Chemogenetic inhibition of dPAG shifted the active defensive responses toward freezing behavior, whereas inhibition of the vlPAG promoted a shift in behavior from passive to active defensive response. SPS model exhibited disrupted behavioral flexibility in defensive responses, with behavioral rigidity when faced with high-intensity threat. This dynamic interaction between the dorsal and ventral parts of PAG may coordinate the capability of behavioral flexibility in defensive responses and provide a potential target for the treatment of diseases concerned with dysfunction of coping strategies to stress-related disorders.

In natural habitats, animals are constantly exposed to diverse threats, and survival depends on the ability to select defensive strategies that are appropriately matched to the perceived intensity and context of danger. Previous work has established various experimental paradigms for both innate and learned defensive behaviors.^7^^,^^29^^,^^30^^,^^31^ Rodents exposed to a rapidly expanding overhead visual stimulus could exhibit immediate flight to shelter,^32^^,^^33^^,^^34^^,^^35^ whereas mice show marked immobility in response to predator-like overhead stimuli.^2^^,^^36^ These visually evoked defensive responses reflect innate behaviors in mice under a naturalistic predatory imminence continuum, while the selection of appropriate defensive strategies can also be shaped through conditioned learning under learned, cue-based perceived threat.^6^^,^^31^^,^^37^^,^^38^ The conditioned defensive behaviors of mice, acquired through learning to associate cues with aversive stimuli, may more accurately model the process by which individuals learn to avoid dangerous events. In our study, we used pure tones with two intensities (60 or 80 dB) as conditioning stimuli to investigate the flexible defensive behavior choices in mice. The different intensities of tones can also be interpreted as representing perceived distal versus proximal threat levels within the predatory imminence continuum. Mice exhibited freezing behavior in response to the lower-intensity tone and escape behavior in response to the higher-intensity tone. Notably, this relationship was not strictly linear across all stimulus combinations. Although the proportion of jumping generally increased with tone and shock intensity, jumping decreased under the highest combined condition (0.9 mA and 80 dB), and was absent when shock intensity was high (0.9 mA) but tone intensity was low (60 dB). This pattern may indicate that active defensive responding does not increase monotonically with perceived threat intensity, but may shift under extreme threat conditions. This framing aligns with ethological models where threat perception is modulated by stimulus salience and perceived proximity to danger. During a Pavlovian conditioning paradigm with a serial compound stimulus, mice typically freeze in response to a tone and flee upon subsequent white noise presentation.^4^ In comparison, pairing foot shocks with tone cues of different intensities provides a simple way to vary cue salience and examine how cue-evoked perceived threat states bias defensive strategy selection. Moreover, the paradigm employed in our study requires only three sessions of fear conditioning to induce the selection of defensive behaviors in mice. Collectively, these findings suggest that animals can select between active and passive defensive strategies consistent with cue-evoked perceived threat modulation. This behavioral paradigm may effectively capture the naturalistic relationship between threat intensity and behavioral output, providing a valuable tool for studying both normal and pathological fear learning.

Appropriate selection of defensive responses relies on specialized and finely tuned neural circuits. Many studies also confirmed that optogenetic activation of different subregions of the PAG can induce different defensive behaviors.^8^^,^^39^^,^^40^ Our study has demonstrated that manipulating activity levels in dPAG and vlPAG is associated with shifts between active and passive defensive responses, supporting the idea that PAG subregions contribute to flexible behavioral selection. In line with prior research, the ultimate choice of defensive responses may depend on interactions between dPAG and vlPAG microcircuits, potentially including mutual inhibitory mechanisms.^8^ Reciprocal inhibition between cells expressing corticotropin-releasing factor and somatostatin-positive neurons in the central amygdala shapes conditioned freezing or flight behavior.^6^ There are also many different subtypes of neurons in PAG, and the role of these neuronal subtypes in regulating flexible choice can be further studied. Our study examined the transformation of defensive behavioral flexibility in response to auditory stimuli of varying intensities, suggesting that neural pathways connected to the auditory-related brain regions may be involved. The PAG is indeed a critical projection site for auditory-related regions,^22^^,^^41^ and how its distinct subregions interact with auditory circuits to influence the selection of flexible defensive behaviors remains an important question. Overall, our findings support an association between PAG subregions and the flexibility of learned defensive behaviors. Moreover, whether specific microcircuits underlie these processes and whether their projections exert distinct functional roles represent key directions for our future investigations.

The ability to flexibly select defensive behaviors enhances survival in healthy individuals, and impairments in this process are commonly observed in psychiatric disease models. Stressed mice display more accelerated defensive responses during innate looming tests, suggesting that stress exposure increases the sensitivity of individuals to avoid threats by changing alertness in the locus coeruleus.^42^ This heightened sensitivity to imminent threat may be a manifestation of hypervigilance in stressed individuals.^43^ In the context of learned defensive behavior, our results show that stress-exposed mice exhibit reduced active responses and increased freezing under high-intensity cues, while responses under low-intensity cues are relatively preserved. While SPS mice also showed reduced locomotor speed in basic control assays, our result suggests that stress exposure may bias defensive response selection, particularly when perceived high threat levels, rather than producing a uniform suppression across all conditions. These stress-induced changes may be reminiscent of heightened vigilance reported in stress-related conditions.^43^ Importantly, although SPS mice showed similar activity in tone habituation, we acknowledge that pain sensitivity and changes in auditory processing could also influence maladaptive flexibility. These factors may collectively contribute to the observed defensive rigidity and should be addressed in future studies. Stress not only affects instinctive behavior in mice but also leads to rigidification of defensive behaviors in PTSD models of learned defensive behavior, and the PAG has been implicated in these studies.^8^^,^^9^^,^^11^^,^^44^ Therefore, further investigation into the roles of PAG-associated pathways in stress-related models may not only provide new insights into the neurobiological basis of flexible learned defensive behavior selection but also identify promising therapeutic targets for correcting maladaptive defensive responses in stress-related psychiatric disorders.

In summary, our study modified tone-cued fear conditioning paradigm reveals that mice can flexibly switch between active and passive defensive responses according to perceived threat conditions, and suggested localized inhibition of PAG subregions as the key mechanism for flexible defensive behavior selection. These findings extend current understanding of how defensive strategies are organized within the PAG and provide a framework for investigating maladaptive defensive responses in animal models of psychiatric disorders.

### Limitations of the study

While our study has extended the understanding of the neural mechanisms underlying defensive behavior selection, there are several limitations. First, we did not distinguish between neuronal subtypes within the dPAG and vlPAG. Therefore, the effects observed here cannot be attributed to specific cell populations. Future studies could benefit from targeting specific neuronal populations to better understand the distinct contributions of these subtypes to defensive behavior. Second, although we used CNO to activate the hM4Di receptors, we did not explore the potential off-target effects or the impact of CNO itself on behavior. We must acknowledge that CNO may influence the observed behaviors independently of the targeted manipulation. Additional experiments should examine the potential confounding effects of CNO administration to clarify its role in our experimental paradigm. Finally, our study was limited to male mice. Given the well-established sex differences in anxiety-related psychiatry,^45^^,^^46^^,^^47^ it will be important to extend this work to both male and female animals in future studies to determine whether the findings are sex-dependent.

## Resource availability

### Lead contact

Further information and requests for resources and reagents should be directed to and will be fulfilled by the lead contact, Guangming Lu (cjr.luguangming@vip.163.com).

### Materials availability

This study did not generate new unique reagents.

### Data and code availability

• All data supporting the findings of this study are available in the article and in the supplemental information and/or from the lead contact upon reasonable request.
• This study did not generate original code.
• Any additional information required to reanalyze the data reported in this study is available from the lead contact upon reasonable request.

## Acknowledgments

This work was supported by grants from the Major Instrument Development Project of 10.13039/501100001809National Natural Science Foundation of China (82127806).

## Author contributions

L.Q., W.W., and G.L. initiated and designed the research. L.Q. and Y.L. wrote the manuscript. L.Q., Y.L., and W.W. performed all the experiments and analyzed the results. R.Q., Z.Z., X.C., J.L., and J.P. contributed to the discussion of the results. L.Z. and G.L. supervised the entire project. All of the authors have discussed and approved the manuscript.

## Declaration of interests

The authors declare no competing interests.

## STAR★Methods

### Key resources table

**Table:** 

REAGENT or RESOURCE	SOURCE	IDENTIFIER
**Antibodies**		

c-Fos antibody	Synaptic Systems	Cat# 226008, RRID: AB_2891278
goat anti-rabbit (Alexa Fluor 488)	Jackson ImmunoResearch Laboratories	Cat# 111-545-003, RRID: AB_2338046

**Bacterial and virus strains**		

rAAV-hSyn-hM4D(Gi)-eYFP-WPRE-hGH pA	BrainVTA(Wuhan) Corp., Ltd	Cat#: PT-0153
AAV-hSyn-eYFP-WPRE-hGH pA	BrainVTA(Wuhan) Corp., Ltd	Cat#: PT-0102

**Chemicals, peptides, and recombinant proteins**		

Clozapine N-oxide (CNO)	MCE	HY-17366
anti-quenching agent with DAPI	Santa Cruz	SC-24941

**Experimental models: Organisms/strains**		

Mouse: C57BL/6J	GemPharmatech	N000013

**Software and algorithms**		

MATLAB	MathWorks	Version R2018, RRID:SCR_001622
Any-Maze	Stoelting Co.	RRID:SCR_014289
ImageJ	National Institutes of Health	RRID:SCR_003070
GraphPad Prism	GraphPad Software	Version 9, RRID:SCR_002798
Adobe Illustrator	Adobe	Version 2026, RRID:SCR_010279

### Experimental model and study participant details

#### Animals

All procedures were approved by the Institutional Animal Care and Use Committee and the Office of Laboratory Animal Resources of Jinling Hospital (protocol no. DZJSXMLS202503190019) and were conducted in accordance with institutional guidelines and relevant national regulations for the care and use of laboratory animals. Wild-type adult male C57BL/6J mice (6–8-week-old) were purchased from GemPharmatech (Nanjing, China). Animals were housed under specific pathogen-free (SPF) conditions on a 12-h light/dark cycle (lights on 06:00–18:00) with *ad libitum* access to food and water. Animals were group-housed (5–6 per cage) in standard cages with bedding under controlled temperature and humidity (22 ± 2 °C, 40–70%). Mice were allowed to acclimate for at least 7 days before experiments. Surgeries and viral injections were performed at 7–9 weeks of age.

### Method details

#### Tone fear conditioning experiment

The fear conditioning paradigm was adapted from the classical Pavlovian fear conditioning protocol. All behavioral sessions were recorded using a USB camera (8 frames/s) connected to the computer, and behavior was analyzed with the ANY-maze software. All animals were acclimated to the behavioral testing room for at least 2 h before each experiment and were returned to the animal facility immediately after testing. On Day 0 (context habituation), mice were placed in a cuboid fear conditioning chamber (Sansbio, 17 cm × 17 cm × 25 cm) with a metal grid floor without any tone presentation. Animals underwent two 4-min habituation sessions, separated by an interval of at least 4 h. On Day 1, mice received a protocol comprising two phases: tone habituation (3 × CS presentations, 30s each, 4 kHz) and fear conditioning (5 × Trials, 29s CS paired with 1s foot shock, 0.6 mA). The CS was set to 80 dB in the high-intensity tone group and 60 dB in the low-intensity tone group by ANY-maze software. The intertrial intervals randomly varied 2-6min. The conditioning chamber was cleaned with 75% ethanol.

To examine the effect of shock intensity, we included four additional groups in which mice received either 0.3 mA or 0.9 mA footshocks under the high-tone (80 dB) or low-tone (60 dB) CS condition. The CS-only control group underwent the same protocol as the experimental group, except that the foot shock was omitted during each fear conditioning trial. And on Day 4 (fear retrieval experiment), the mice were re-exposed to an altered context enclosed by a white acrylic cylinder (18 cm diameter), which differed from the original conditioning context. The retrieval protocol consisted of 10 CS presentations (identical to the CS on Day1, 30 s each) with 90 s intertrial intervals. The retrieval context was wiped down with 1% acetic acid.

#### Open-field test (OFT)

The open-field test was performed in an opaque plexiglas arena (50 × 50 × 38 cm) under 200 lux illumination. The central quadrant, defined as the innermost 25% of total arena area (25 × 25 cm), was automatically demarcated using ANY-maze software. After 30-min acclimation in the behavioral testing room, each mouse was gently positioned in the arena center and allowed 5-min free exploration. All locomotor activities were recorded by an overhead video tracking system for subsequent analysis of total distance moved and center duration.

#### Elevated plus maze (EPM)

The elevated plus maze (elevated 50 cm above floor level) consisted of two open arms (35 cm × 5 cm) and two closed arms (35 cm × 5 cm × 15 cm) intersecting at right angles. After 30-min acclimation in the behavioral testing room, mice were placed in the central zone (10 × 10 cm) facing an open arm and allowed a 5-min free exploration period under 200 lux illumination. All trials were recorded by an overhead camera connected to ANY-maze software for subsequent analysis of time spent in open arms and the number of entries.

#### Chemogenetics

For the manipulation of neurons in the subregion of PAG, respectively, 70 nL of AAV virus rAAV-hSyn-hM4D (Gi)-eYFP-WPRE-hGH pA, AAV-hSyn-eYFP-WPRE-hGH pA (BrainVTA Co., Ltd, Wuhan, China) was bilaterally injected into the dPAG(AP –4.65 mm; ML ±0.42 mm; DV –2.50 mm) and vlPAG(AP –4.72 mm; ML ±0.45 mm; DV –2.80 mm) in mice to silence the local brain neurons. Three weeks later, Clozapine-N-oxide (CNO, MCE, Shanghai, China, 2 mg/kg) was injected intraperitoneally 30 min before the behavior testing to activate the hM4Di receptor. The virus injection site was verified, and data from mice with incorrect sites or unilateral expression were excluded.

#### Histology

The mice were deeply anesthetized with isoflurane and transcardially perfused with Phosphate-Buffered Saline (PBS, 0.01 M), followed by 4% paraformaldehyde (PFA). The brains were removed and post-fixed in 4% paraformaldehyde overnight at 4°C and then cryoprotected with 30% sucrose in PBS for 2 days at 4°C. The processed tissue was sectioned into serial slices through the relevant regions at 40 μm by a cryostat (Leica CM1950). Brain sections spanning the PAG along the anterior–posterior axis (bregma −4.16 to −4.84, according to the atlas) were collected from each mouse. To verify viral expression, sections were coverslipped with a DAPI-containing mounting medium (Santa, sc-24941) and imaging was performed using constant parameters on a Leica TCS SP8 confocal laser scanning microscope. Mice with unilateral viral expression or incorrect targeting were excluded from further analyses. For each mouse, three representative PAG sections (bregma −4.24 mm, −4.48 mm, and −4.72 mm) were selected along the anterior–posterior axis. The viral expression area was manually delineated on each section, and the eYFP-positive area fraction within each PAG subregion ROI was quantified in ImageJ to obtain area-based regional transduction coverage. Expression maps were then overlaid across mice within each group to confirm accurate targeting of the intended PAG subregion.

#### C-Fos immunostaining

The c-Fos immunostaining sections were prepared as described in the histology. The sections were washed three times for 10 min each in PBS (0.01 M), and then permeabilized with 0.3% Triton X-100 for 30 min, followed by blocking with 2% BSA at room temperature for 1.5 h. The slices were then incubated with c-Fos antibody (1:500, Synaptic Systems, 226008) at 4°C for 24 h. After washing, sections were conjugated with an Alexa 488–conjugated secondary antibody (goat anti-rabbit, 1:500, Jackson Immuno) for 2 h at room temperature. The nuclei were stained with DAPI (Santa, sc-24941), and imaging was performed using constant parameters on a Leica TCS SP8 confocal laser scanning microscope. All confocal acquisition settings (e.g., laser power, gain, pinhole, and exposure/scan speed) were kept identical across samples. Lastly, for each mouse, three coronal sections containing the PAG were selected at approximately bregma −4.24 mm, −4.48 mm, and −4.72 mm (as defined in the mouse brain atlas), thereby covering a consistent rostro–caudal range of the PAG across animals. PAG subregions were delineated manually according to standard anatomical boundaries, and Fos-positive neurons were counted bilaterally (left and right) in each subregion. The sections were adjusted to the same contrast by ImageJ across different groups before cell counting quantification. All quantifications were performed blind to experimental conditions. The mean Fos count across the three sections (averaged per animal) was used as the final value for statistical analysis. For c-Fos analysis, three groups were included. The SPS mice underwent stress and subsequently received tone–shock conditioning, while the C57 mice received tone–shock conditioning without prior stress exposure. The CTR (CS-only) group was exposed to the tone alone, without SPS or shock. The CTR (CS-only) group was used as the reference and set to 100%. Values from each experimental group were normalized to the CTR baseline and expressed as % of the control.

#### Single prolonged stress (SPS) model

Male mice were randomly assigned to either the SPS group or the control (CTR) group. The SPS protocol was conducted on Day 0, following established procedures with slight modifications. First, mice were subjected to restraint stress by placing them individually in well-ventilated restrainers for 2 h. Immediately following restraint, they were exposed to forced swimming for 20 min in a cylindrical container filled with water maintained at 25 ± 1°C and a depth of approximately 20 cm to prevent escape. After a brief recovery period of 10 min, the animals were then exposed to isoflurane anesthesia (2–3% in oxygen) for 5 min to simulate loss of consciousness and enhance stress impact. Control mice were handled to contact the same environment but not subjected to SPS procedures. Following the SPS session, mice were returned to their home cages for the fear conditioning experiment.

#### Behavioral analysis

Freezing time, freezing score, and movement speed were automatically calculated using ANY-maze software. Data from the CS (29 s) and Pre-CS (30 s) time periods were extracted from each mouse for subsequent analysis. Other defensive behavioral metrics, including jumping, darting,^31^ motion and freezing time during the last two CS presentations (trial 4 and 5), were manually classified by two blinded observers based on the video recordings. Freezing was defined as a complete lack of movement, except for respiration (immobility for ≥500 ms and freezing score <10).^48^ Jumps were defined as the period during which the mouse had all four paws above the chamber floor. Darts were defined as a rapid, short-distance horizontal movement triggered by sudden threats, often combined with freezing or risk assessment (most of the freezing scores>250, but excluding the jump behavior).^47^ The motion refers to the simple physical activity within the fear conditioning box, excluding freezing and darting behaviors (typically freezing score ranging from 1 to 250). An escape score was computed for each animal as the ratio of its average freezing score during CS presentation (29 s) to its average pre-CS speed (30 s),^4^^,^^31^ with additional points added for escape-like behaviors, according to the following formula: Escape Score = (Speed _ave CS_/Speed _ave pre-CS_) + 1 × (Number of Dart) + 2 × (Number of Jump).

### Quantification and statistical analysis

All data are presented as mean ± SEM. Statistical analyses were performed using GraphPad Prism 9 (GraphPad Software, San Diego, CA). Date distribution was assessed using the Shapiro-Wilk normality test (α = 0.05). For experiments involving two factors, such as group and trial, data were analyzed using two-way repeated-measures ANOVA followed by Bonferroni’s multiple-comparisons test, as appropriate. For comparisons between two groups, unpaired two-tailed Student’s t tests were used for normally distributed data, whereas Mann–Whitney U tests were used for data that did not meet normality assumptions. Differences were significant with a *p* value <0.05. Exact *n* values, the definition of n, the statistical tests used for each experiment, and significant information are provided in the figure legends and Results section.

#### Additional resources

No additional resources were generated in this study.

## Supplemental information

Document S1. Figures S1–S5 and Table S2

Table S1. Shapiro-Wilk normality test results and two-way repeated-measures ANOVA effect sizes across groups

Table S3. Normality test results and parametric/nonparametric group comparisons with effect sizes across groups
