# Supplementary material for: PAG-dependent defensive strategy selection: From stress-induced rigidity to adaptive flexibility
Source: iScience. 2026 Apr 29;29(10):115927. doi: 10.1016/j.isci.2026.115927 (PMC13626930; doi:10.1016/j.isci.2026.115927)
Supplement: Supplementary file 1 [file mmc1.pdf]

iScience, Volume *29*

Supplemental information

PAG-dependent defensive strategy selection: From stress-induced rigidity to adaptive flexibility

Lianli Qiu, Yueqin Liu, Rongfeng Qi, Zhiqiang Zhang, Xiaoqing Cheng, Jianrui Li, Jing Pan, Wei Wu, Longjiang Zhang, and Guangming Lu

Supplemental Figures

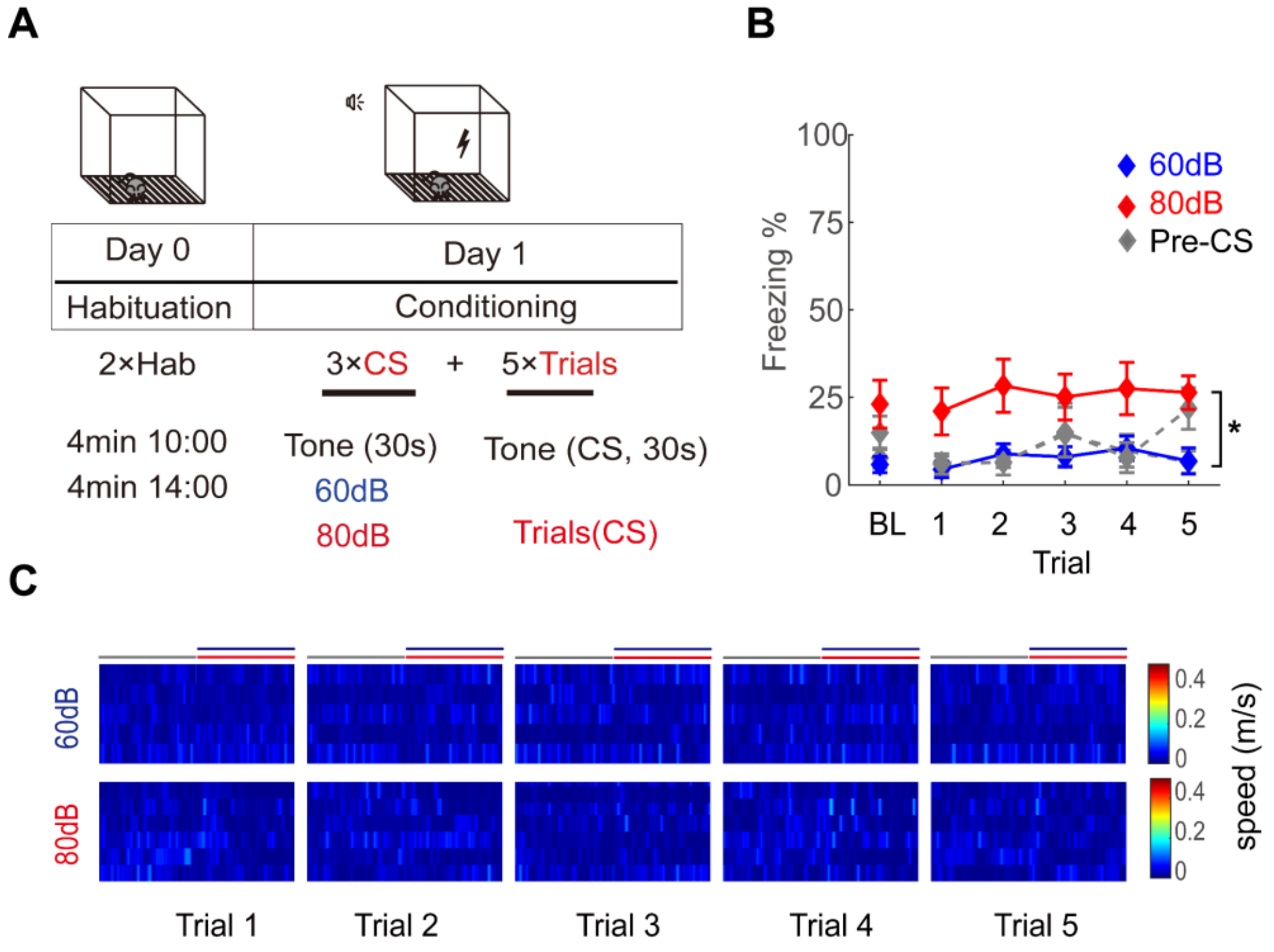

Figure S1. Distinctive tone exposure alone does not evoke different defensive responses

(A) Schematic of the control experimental paradigm in which mice were exposed to pure tones paired with no shock (60 dB, n = 5 mice, 80 dB, n = 6 mice).

(B) Freezing percentage in mice exposed to 60 dB or 80 dB pure tones. Normality was assessed using Shapiro–Wilk tests (Table S1). Two-way repeated-measures ANOVA (Trial×dB) revealed significant Trial×dB interactions for freezing percentage ($F(4,36) = 0.0684$, $P = 0.9911$), with significant main effects of dB (freezing percentage, $F(1,9) = 8.2700$, $P = 0.0183$). Bonferroni-corrected simple-effects comparisons are provided in Table S2.

(C) Representative heatmaps showing individual mouse movement speed during exposure to tones.

Data are presented as mean ± SEM, * $p < 0.05$.

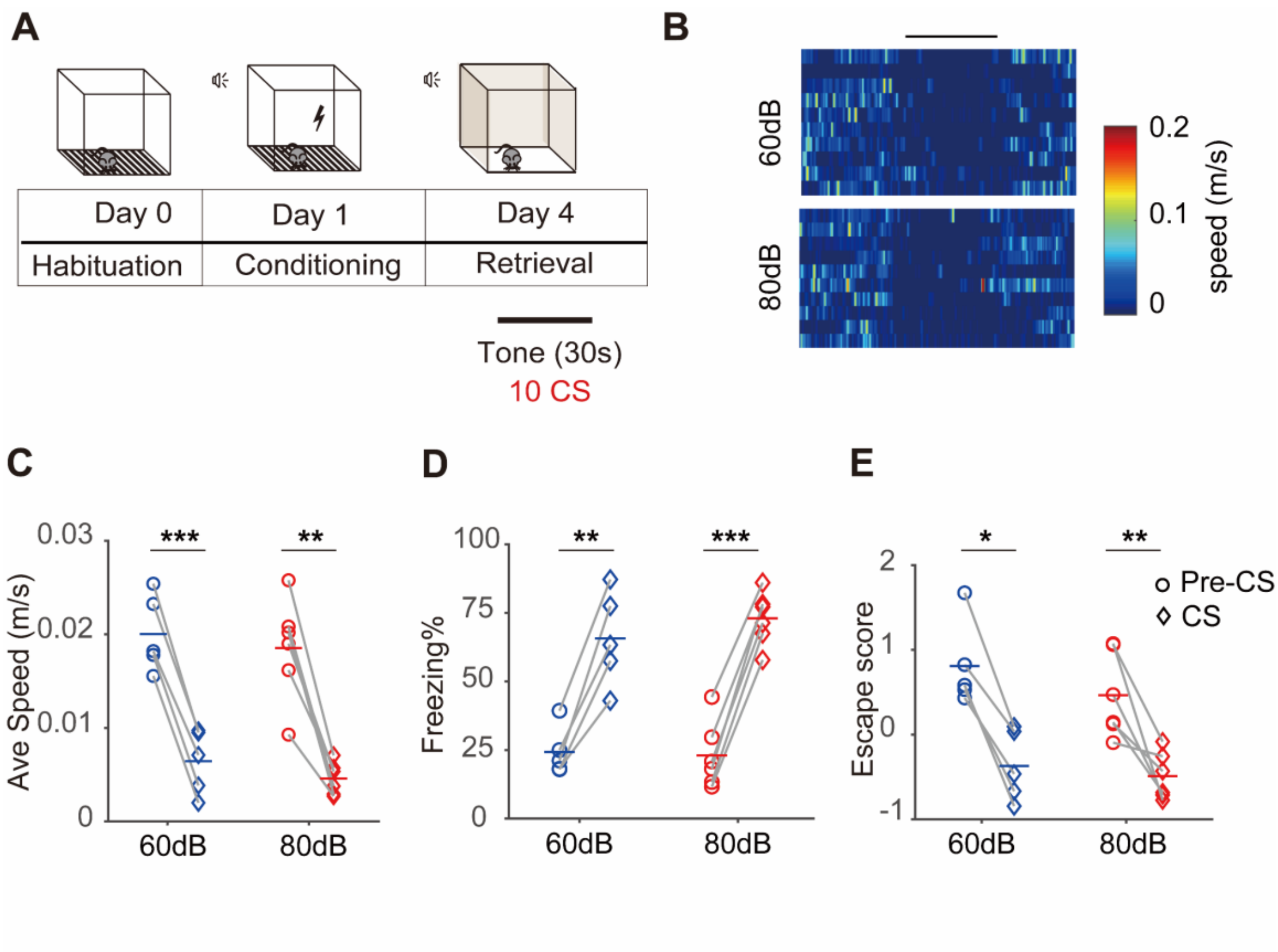

Figure S2. Passive defensive response was evoked by distinctive tone intensities during fear memory retrieval.

(A) Schematic of fear memory retrieval, in which mice were re-exposed to 10 conditioned stimuli (CS, 30 s) at either 60 dB or 80 dB.

(B) The representative heatmaps of the movement speed during CS presentation under fear-conditioned retrieval.

(C-E) Quantification of average speed, freezing percentage, and escape score during Pre-CS and CS retrieval sessions at 60 dB and 80 dB tone intensities (60 dB, n = 5 mice, 80 dB, n = 6 mice). Normality was assessed using Shapiro–Wilk tests (Table S3). Average speed, freezing percentage and escape score were compared using paired t-test. Normality and paired group comparison of different groups was provided in Table S3.

Data are presented as mean ± SEM, * $p < 0.05$, ** $p < 0.01$, *** $p < 0.001$.

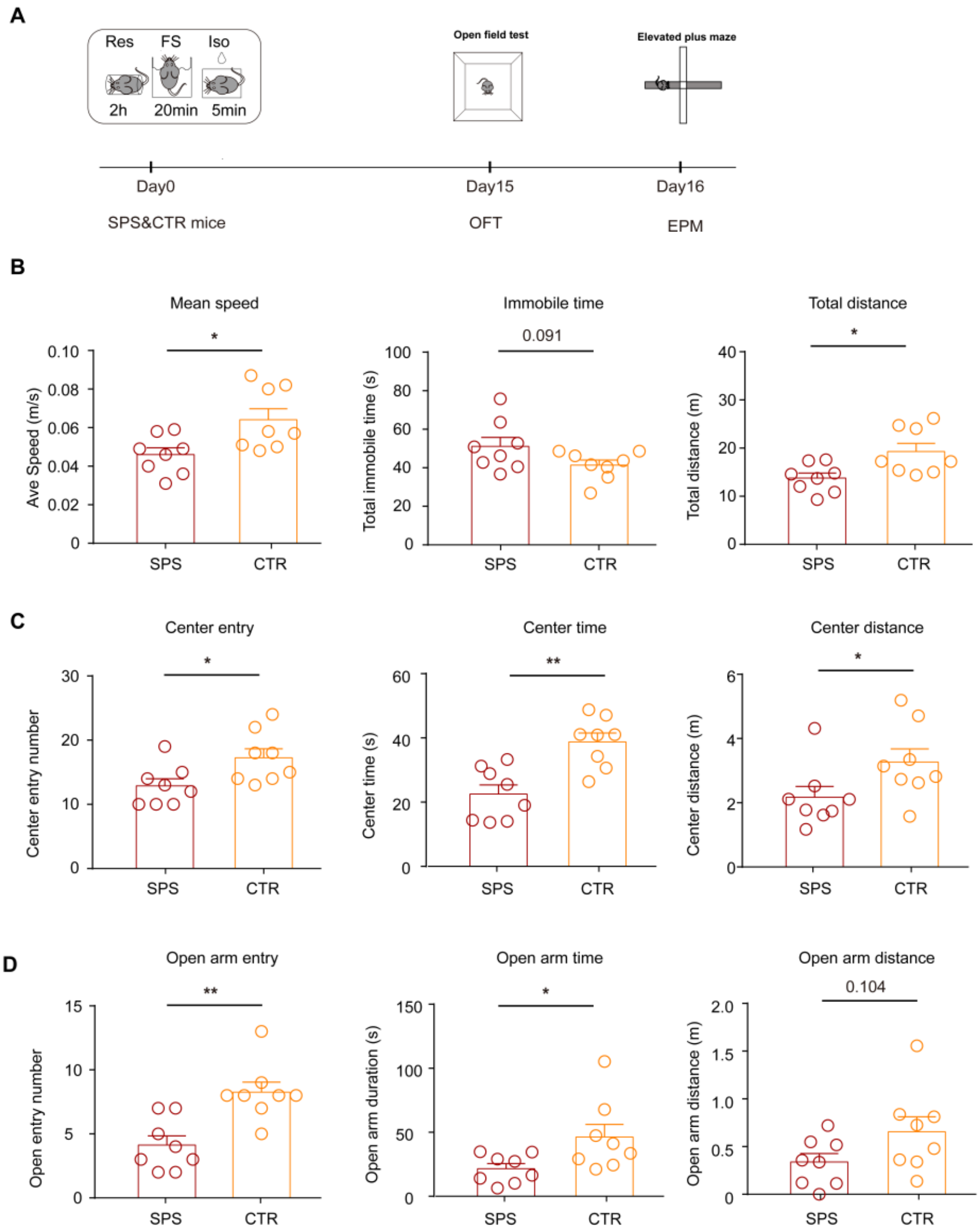

Figure S3. Basic control behavioral assays of the OFT and EPM in SPS and control mice

(A) Experimental design for the SPS and control mice.

(B) For the two mouse groups, anxiety-related movement was assessed based on (left-right): mean speed, immobile time, and total distance in OFT (SPS mice, n = 8 mice, CTR mice, n = 8 mice). All groups were compared using two-tailed Welch's t-tests (mean speed, $p = 0.019$, immobile time, $p = 0.091$, total distance, $p = 0.019$).

(C) For the two mouse groups, anxiety-related movement was assessed based on (left-right): the number of center entries, time in center zone, and center distance in OFT. Center entry number and center time were compared using two-tailed Welch's t-tests (center entry number, $p = 0.03$, center time, $p = 0.001$). Center distance was compared using the Mann–Whitney U test ($p = 0.038$).

(D) For the two mouse groups, anxiety-related movement was assessed based on (left-right): the number of entries into the open arm, time in open arm, and open arm distance in EPM. All groups were compared using two-tailed Welch's t-tests (open arm entry, $p = 0.002$, open arm time, $p = 0.046$, open arm distance, $p = 0.104$).

Normality was assessed using Shapiro–Wilk tests (Table S3). Data are presented as mean ± SEM, $^{*}p < 0.05$, $^{**}p < 0.01$.

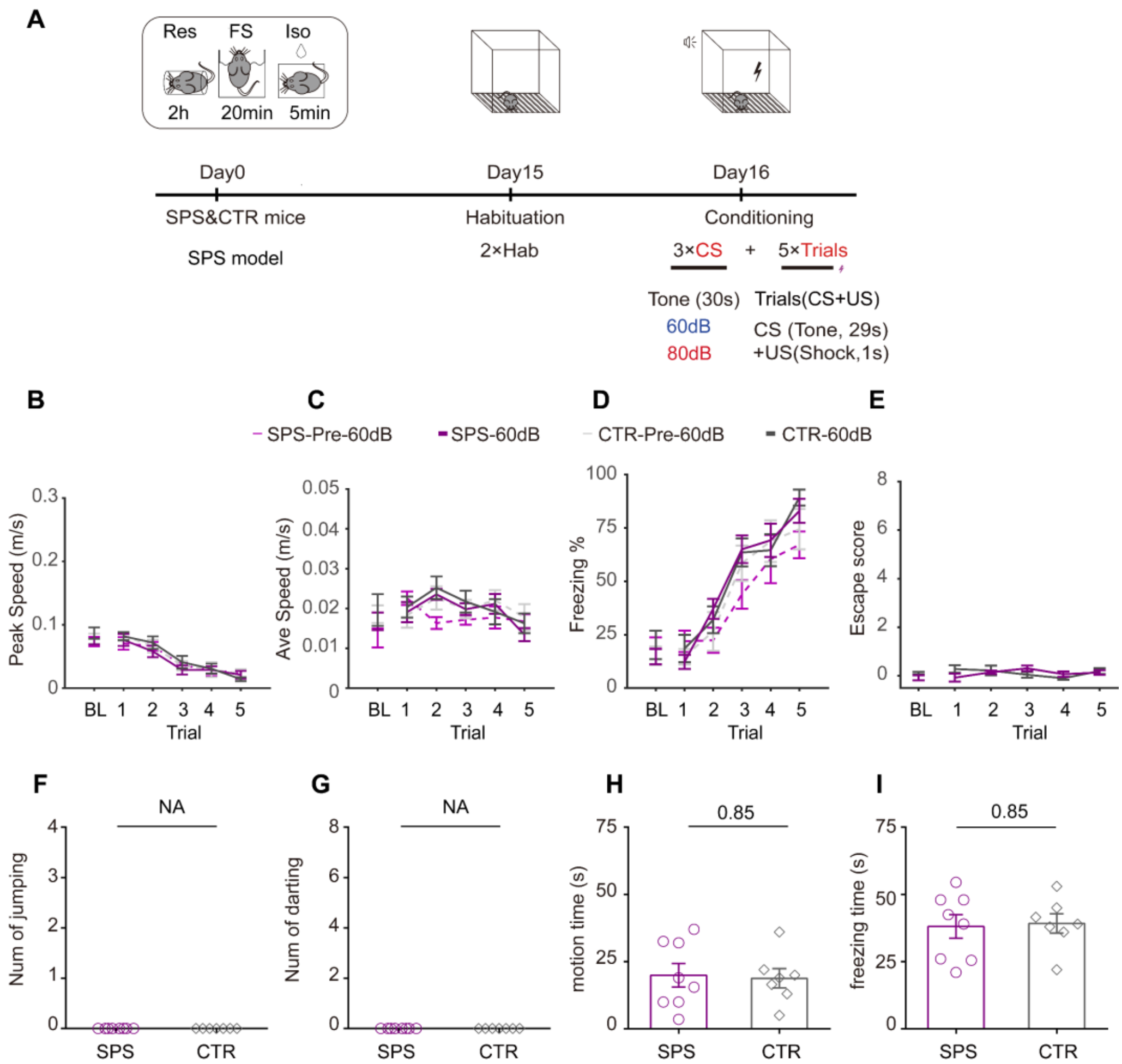

Figure S4. Stress exposure did not affect the passive defensive responses when faced with low-tone intensity threat

(A) Experimental design for the SPS and control mice exposed to the low tone intensity fear conditioning (CS, 60 dB, US, 0.6 mA).

(B-E) Quantification of peak speed, average speed, freezing percentage, and escape score during baseline and five consecutive trials exposed to 60 dB in SPS mice (n = 8 mice) and control mice (n = 7 mice). Normality was assessed using Shapiro–Wilk tests (Table S1). Two-way repeated-measures ANOVA (Trial×SPS) revealed significant Trial×SPS interactions for peak speed ($F(4,52) = 1.1980$, $P = 0.3229$), average speed ($F(4,52) = 0.8030$, $P = 0.5287$), freezing percentage ($F(4,52) = 0.5750$, $P = 0.6820$), and escape score ($F(4,52) = 2.0060$, $P = 0.1073$), with significant main effects of SPS (peak speed, $F(1,13) = 0.3780$, $P = 0.5493$, average speed, $F(1,13) = 0.0751$, $P = 0.7883$, freezing percentage, $F(1,13) = 0.1003$, $P = 0.7565$, escape score, $F(1,13) = 0.0150$, $P = 0.9043$). Bonferroni-corrected simple-effects comparisons are provided in Table S2.

(F-I) Behavioral metrics of jumping, darting, motion, and freezing times were manually quantified during the last two CS presentations of SPS and CTR mice. Normality was assessed using Shapiro–Wilk tests (Table S3). Jumping and darting showed no variation (all values = 0) in both groups, and motion and freezing times were compared using two-tailed Welch's t-tests (motion time, $p = 0.8450$, freezing time, $p = 0.8450$).

Data are presented as mean ± SEM.

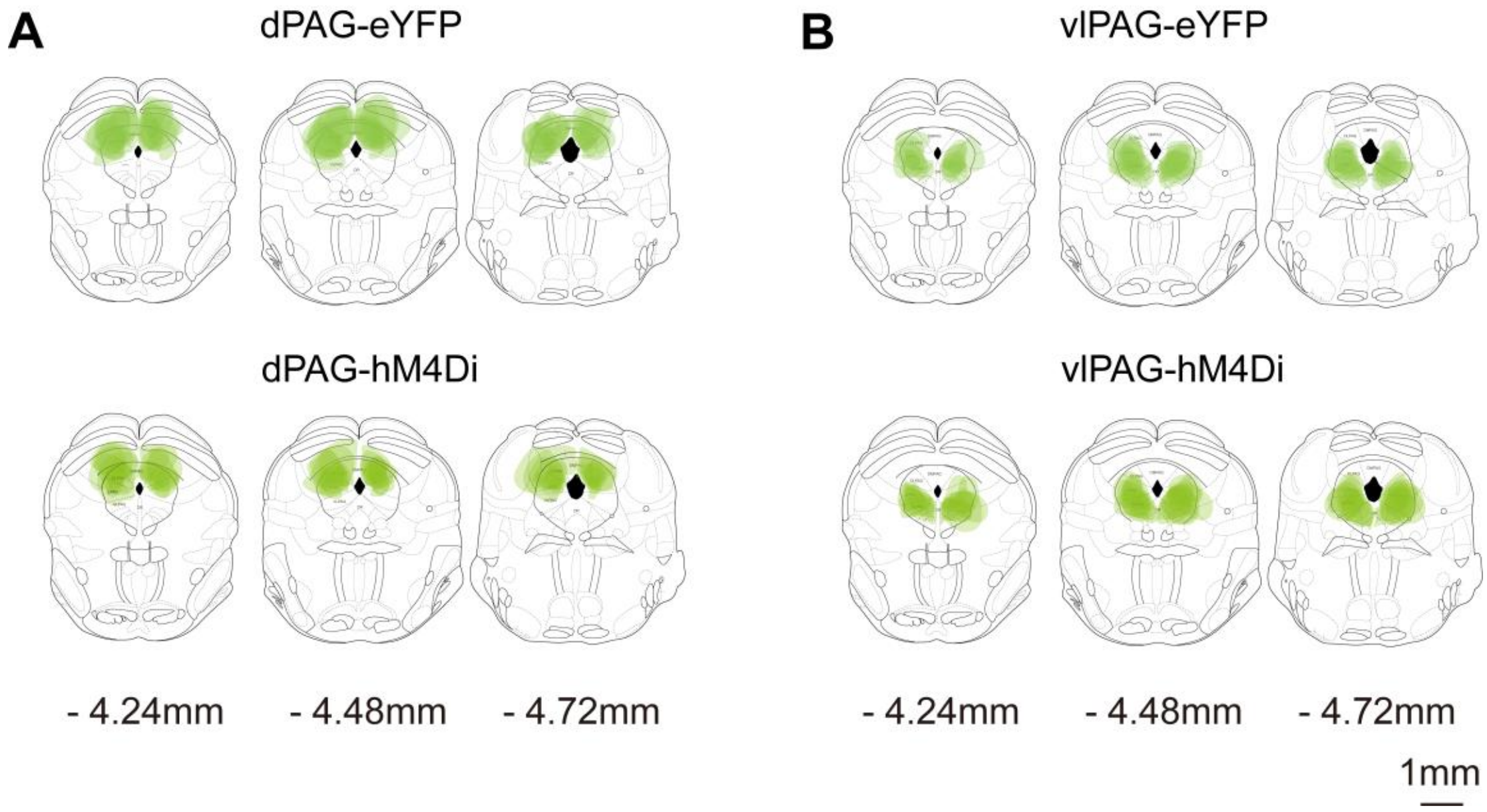

Figure S5. Viral expressions in all different experimental groups.

(A) Extent of eYFP and hM4Di viral expression in dPAG groups (dPAG-eYFP = 66.9 ± 4.7%, n = 8 mice, dPAG-hM4Di = 56.0 ± 4.4%, n = 9 mice). The quantification of target dPAG was calculated as the area-based coverage within each target region. Coverage dPAG (%) = (eYFP/hM4Di-positive area within dPAG / total dPAG area) × 100, measured bilaterally on the three A–P levels (Bregma -4.24, -4.48, -4.72) and averaged to yield one value per mouse.

(B) Extent of eYFP and hM4Di viral expression in vlPAG groups (vlPAG-eYFP = 60.8 ± 3.8%, n = 7 mice), vlPAG-hM4Di = 63.8 ± 2.9%, n = 9 mice). The quantification of target vlPAG was calculated as the area-based coverage within each target region. Coverage vlPAG (%) = (eYFP/hM4Di-positive area within vlPAG / total vlPAG area) × 100, measured bilaterally on the three A–P levels (Bregma -4.24, -4.48, -4.72) and averaged to yield one value per mouse.

Table S2. Two-way ANOVA Across Groups with Multiple-Comparison Adjustment (Bonferroni's multiple comparison test)

Figure	trial1	trial2	trial3	trial4	trial5
Figure1C	ns	0.9728	< 0.0001	< 0.0001	< 0.0001
Figure1D	ns	ns	0.0002	< 0.0001	< 0.0001
Figure1E	0.3472	0.4954	0.1119	< 0.0001	< 0.0001
Figure1F	ns	ns	0.0011	< 0.0001	0.0003
Figure2C	ns	ns	0.7348	0.0276	0.1089
Figure2D	ns	ns	0.77	0.0014	0.0075
Figure2E	ns	ns	ns	< 0.0001	0.0048
Figure2F	ns	ns	ns	0.0007	0.0729
Figure3E	ns	ns	0.0243	0.0131	0.0010
Figure3F	ns	ns	0.031	0.0186	0.0022
Figure3G	ns	ns	0.0009	0.0128	0.0013
Figure3H	ns	ns	0.8244	0.0212	0.0172
Figure4C	< 0.0001	0.1545	0.0014	ns	0.0214
Figure4D	< 0.0001	0.0005	0.0053	ns	0.2205
Figure4E	0.2492	0.0106	< 0.0001	< 0.0001	< 0.0001
Figure4F	0.0013	ns	ns	0.5728	0.1298
Figure S1B	0.2066	0.0888	0.1819	0.1874	0.0888
Figure S4B	ns	ns	ns	ns	ns
Figure S4C	ns	ns	ns	ns	ns
Figure S4D	ns	ns	ns	ns	ns
Figure S4E	0.2477	ns	0.7072	ns	ns

Note: p＞0.0999 was reported as *ns.
